# *O*-Propyl *N*-phenyl­thio­carbamate

**DOI:** 10.1107/S160053681202140X

**Published:** 2012-05-19

**Authors:** Panyapon Sudkaow, Chien Ing Yeo, Seik Weng Ng, Edward R. T. Tiekink

**Affiliations:** a Department of Chemistry, University of Malaya, 50603 Kuala Lumpur, Malaysia; b Department of Chemistry, Faculty of Science, Prince of Songkla University, Hat-Yai, Thailand 90110; c Chemistry Department, Faculty of Science, King Abdulaziz University, PO Box 80203 Jeddah, Saudi Arabia

## Abstract

Two independent mol­ecules comprise the asymmetric unit in the title thio­carbamide derivative, C_10_H_13_NOS. These differ in the relative orientations of terminal ethyl groups [C—C—C—O torsion angles = −66.95 (13) and 55.92 (13)°, respectively]. The phenyl ring is twisted out of the plane of the central residue [C_q_—N—C_ph_—C_ph_ = −146.20 (12) and −144.15 (12)°, respectively; q = quaternary and ph = phen­yl]. The independent mol­ecules are linked into a dimeric aggregate by N—H⋯S hydrogen bonds and an eight-membered thio­amide {⋯H—N—C=S}_2_ synthon.

## Related literature

For related thio­carbamaide structures, see: Ho *et al.* (2005 ▶); Kuan *et al.* (2007 ▶).
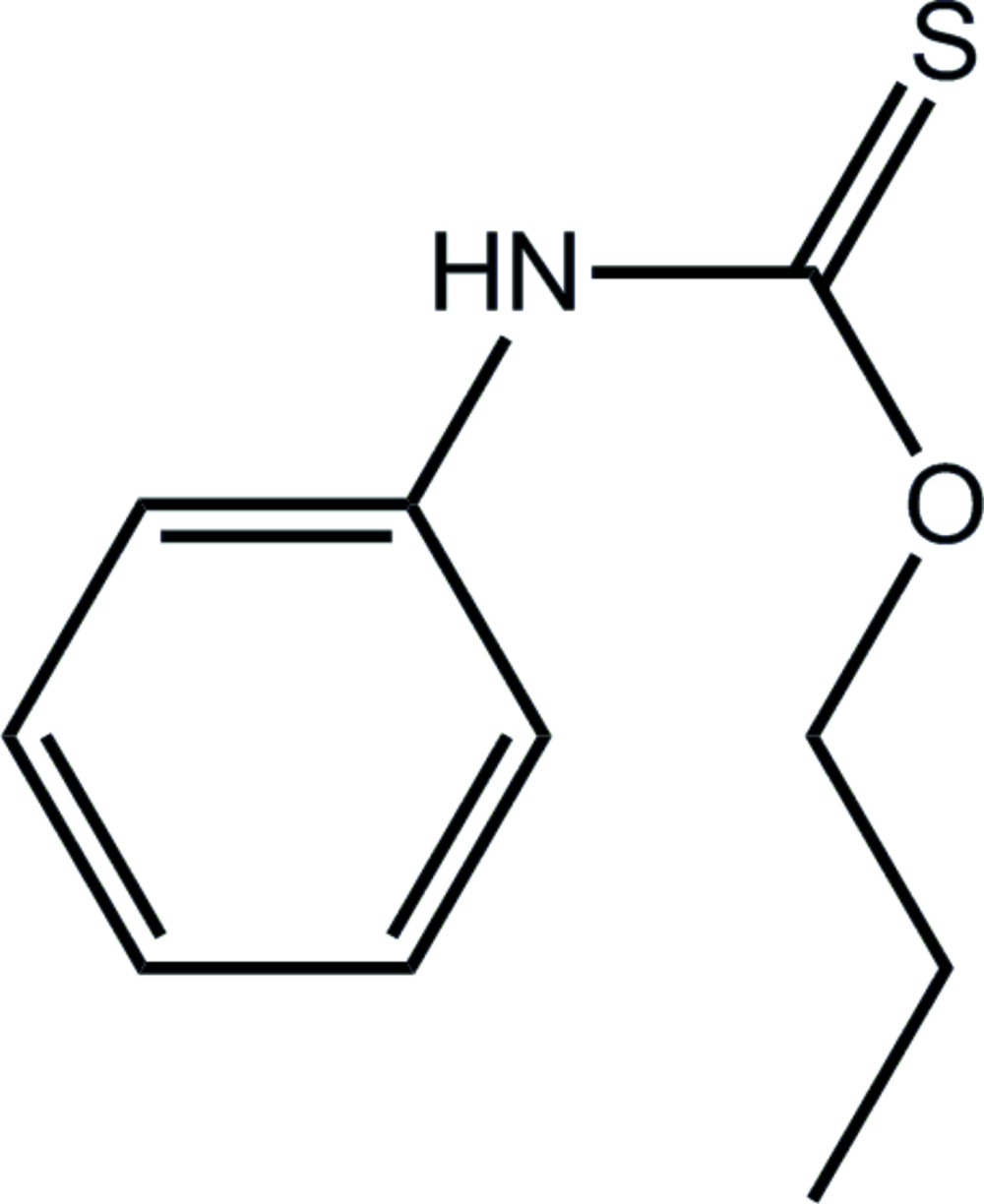


## Experimental

### 

#### Crystal data

C_10_H_13_NOS
*M* *_r_* = 195.27
Triclinic, 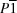

*a* = 8.9230 (4) Å
*b* = 9.8752 (4) Å
*c* = 12.9613 (5) Å
α = 98.037 (3)°
β = 105.866 (4)°
γ = 104.533 (4)°
*V* = 1036.46 (7) Å^3^
*Z* = 4
Cu *K*α radiation
μ = 2.45 mm^−1^
*T* = 100 K
0.35 × 0.30 × 0.25 mm

#### Data collection

Agilent SuperNova Dual diffractometer with an Atlas detector
Absorption correction: multi-scan (*CrysAlis PRO*; Agilent, 2011 ▶) *T*_min_ = 0.772, *T*_max_ = 1.000
7531 measured reflections
4249 independent reflections
4049 reflections with *I* > 2σ(*I*)
*R*_int_ = 0.014

#### Refinement

*R*[*F* ^2^ > 2σ(*F* ^2^)] = 0.030
*wR*(*F* ^2^) = 0.081
*S* = 1.04
4249 reflections
243 parameters
2 restraints
H atoms treated by a mixture of independent and constrained refinement
Δρ_max_ = 0.22 e Å^−3^
Δρ_min_ = −0.26 e Å^−3^

### 

Data collection: *CrysAlis PRO* (Agilent, 2011 ▶); cell refinement: *CrysAlis PRO*; data reduction: *CrysAlis PRO*; program(s) used to solve structure: *SHELXS97* (Sheldrick, 2008 ▶); program(s) used to refine structure: *SHELXL97* (Sheldrick, 2008 ▶); molecular graphics: *ORTEP-3* (Farrugia, 1997 ▶), *DIAMOND* (Brandenburg, 2006 ▶) and *QMol* (Gans & Shalloway, 2001 ▶); software used to prepare material for publication: *publCIF* (Westrip, 2010 ▶).

## Supplementary Material

Crystal structure: contains datablock(s) global, I. DOI: 10.1107/S160053681202140X/hg5228sup1.cif

Structure factors: contains datablock(s) I. DOI: 10.1107/S160053681202140X/hg5228Isup2.hkl

Supplementary material file. DOI: 10.1107/S160053681202140X/hg5228Isup3.cml

Additional supplementary materials:  crystallographic information; 3D view; checkCIF report

## Figures and Tables

**Table 1:** Hydrogen-bond geometry (Å, °)

*D*—H⋯*A*	*D*—H	H⋯*A*	*D*⋯*A*	*D*—H⋯*A*
N1—H1*n*⋯S2	0.88 (1)	2.51 (1)	3.3667 (10)	164 (2)
N2—H2*n*⋯S1	0.88 (1)	2.52 (1)	3.3765 (10)	167 (2)

**Fig. 1.**
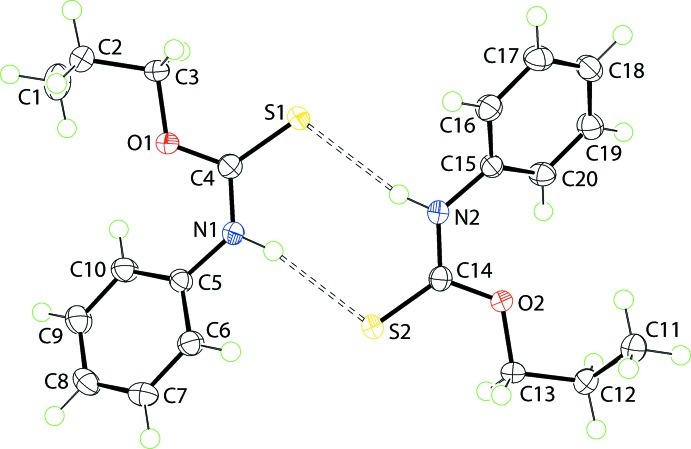
The molecular structures of the two independent molecules comprising (I) showing the atom-labelling scheme and displacement ellipsoids at the 70% probability level. The dashed lines represent N—H hydrogen bonds.

**Fig. 2.**
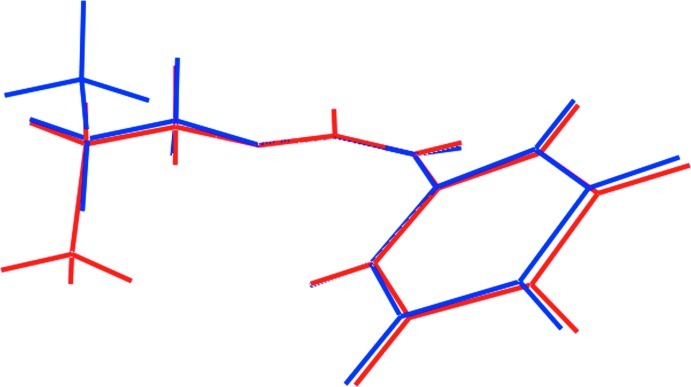
Overlay diagram of the two independent molecules comprising the asymmetric unit of (I). The first independent molecule (with the S1 atom) is shown in red and the second (S2) in blue. The S, O and N atoms in each molecule have been overlapped.

## supplementary crystallographic information

### Comment

The title thiocarbamate, (I), was investigated as a continuation of systematic evaluation of the structural features of this class of compound (Ho *et al.*, 2005; Kuan *et al.*, 2007). In (I), Fig. 1, two independent molecules comprise the asymmetric unit, and these have very similar molecular conformations as seen in the overlay diagram, Fig. 2. The difference arises in the pseudo mirror relationship in the terminal ethyl group as seen in the C1—C2—C3—O1 and C11—C12—C13—O2 torsion angles of -66.95 (13) and 55.92 (13)°, respectively. A further twist is evident in the molecule as seen in the value of the C4—N1—C5—C6 torsion angle of -146.20 (12)°; the equivalent torsion angle for the second independent molecule [C14—N2—C15—C16] is -144.15 (12)°. The geometric parameters match literature precedents (Ho *et al.*, 2005; Kuan *et al.*, 2007)

The independent molecules are linked into a dimeric aggregate by N—H···S hydrogen bonds and an eight-membered thioamide {···H—N—C=S}_2_ synthon, Table 1, as found in most thiocarbamate structures (Kuan *et al.*, 2007). Molecules assemble into a three-dimensional architecture with no specific interactions between them.

### Experimental

Phenyl isothiocyanate (2 ml) was added drop-wise to a stirred solution of NaOH (1 mol equiv.) in *n*-propanol (20 ml) and stirred for 3 h. Excess HCl (5*M*) was then added and the solution was stirred for a further 1 h. The product was then extracted with CHCl_3_ and left for evaporation at room temperature, yielding colourless crystals after 2 weeks. IR (KBr, cm^-1^): ν(N—H) 3211 (br); ν(C—N) 1410 (*s*); ν(C═S) 1221 (*s*); ν(C—O) 1060 (*s*). ^1^H NMR (CDCl_3_, p.p.m.): δ 1.00 (t, J = 7.44 Hz, 3H, CH_3_), 1.80 (sextet, J = 7.06 Hz, 2H, CH_2_), 4.54 (br, 2H, CH_2_O), 7.16 – 7.35 (br, m, 5H aryl-H), 8.85 (br, 1H NH). *M*.pt: 311–312 K.

### Refinement

Carbon-bound H-atoms were placed in calculated positions [C—H = 0.95 to 0.99 Å, *U*_iso_(H) = 1.2–1.5*U*_eq_(C)] and were included in the refinement in the riding model approximation. The amino-H atoms were refined with the distance restraint N—H = 0.88±0.01 Å.

### Crystal data

**Table:**

C_10_H_13_NOS	*Z* = 4
*M**_r_* = 195.27	*F*(000) = 416
Triclinic, *P*1	*D*_x_ = 1.251 Mg m^−^^3^
Hall symbol: -P 1	Cu *K*α radiation, λ = 1.54184 Å
*a* = 8.9230 (4) Å	Cell parameters from 5287 reflections
*b* = 9.8752 (4) Å	θ = 4.7–76.4°
*c* = 12.9613 (5) Å	µ = 2.45 mm^−^^1^
α = 98.037 (3)°	*T* = 100 K
β = 105.866 (4)°	Prism, colourless
γ = 104.533 (4)°	0.35 × 0.30 × 0.25 mm
*V* = 1036.46 (7) Å^3^	

### Data collection

**Table:**

Agilent SuperNova Dual diffractometer with an Atlas detector	4249 independent reflections
Radiation source: SuperNova (Cu) X-ray Source	4049 reflections with *I* > 2σ(*I*)
Mirror monochromator	*R*_int_ = 0.014
Detector resolution: 10.4041 pixels mm^-1^	θ_max_ = 76.6°, θ_min_ = 4.8°
ω scan	*h* = −7→11
Absorption correction: multi-scan (*CrysAlis PRO*; Agilent, 2011)	*k* = −11→12
*T*_min_ = 0.772, *T*_max_ = 1.000	*l* = −15→16
7531 measured reflections	

### Refinement

**Table:**

Refinement on *F*^2^	Primary atom site location: structure-invariant direct methods
Least-squares matrix: full	Secondary atom site location: difference Fourier map
*R*[*F*^2^ > 2σ(*F*^2^)] = 0.030	Hydrogen site location: inferred from neighbouring sites
*wR*(*F*^2^) = 0.081	H atoms treated by a mixture of independent and constrained refinement
*S* = 1.04	*w* = 1/[σ^2^(*F*_o_^2^) + (0.0468*P*)^2^ + 0.325*P*] where *P* = (*F*_o_^2^ + 2*F*_c_^2^)/3
4249 reflections	(Δ/σ)_max_ = 0.001
243 parameters	Δρ_max_ = 0.22 e Å^−^^3^
2 restraints	Δρ_min_ = −0.26 e Å^−^^3^

### Special details

**Table:**

Geometry. All e.s.d.'s (except the e.s.d. in the dihedral angle between two l.s. planes) are estimated using the full covariance matrix. The cell e.s.d.'s are taken into account individually in the estimation of e.s.d.'s in distances, angles and torsion angles; correlations between e.s.d.'s in cell parameters are only used when they are defined by crystal symmetry. An approximate (isotropic) treatment of cell e.s.d.'s is used for estimating e.s.d.'s involving l.s. planes.
Refinement. Refinement of *F*^2^ against ALL reflections. The weighted *R*-factor *wR* and goodness of fit *S* are based on *F*^2^, conventional *R*-factors *R* are based on *F*, with *F* set to zero for negative *F*^2^. The threshold expression of *F*^2^ > σ(*F*^2^) is used only for calculating *R*-factors(gt) *etc*. and is not relevant to the choice of reflections for refinement. *R*-factors based on *F*^2^ are statistically about twice as large as those based on *F*, and *R*- factors based on ALL data will be even larger.

### Fractional atomic coordinates and isotropic or equivalent isotropic displacement parameters (Å^2^)

**Table:**

	*x*	*y*	*z*	*U*_iso_*/*U*_eq_	
S1	0.71820 (3)	0.96137 (3)	0.53434 (2)	0.01838 (9)	
S2	0.28880 (3)	0.57145 (3)	0.43767 (2)	0.01822 (9)	
O1	0.69183 (10)	1.01848 (8)	0.33618 (6)	0.01621 (17)	
O2	0.32823 (10)	0.50276 (9)	0.63279 (7)	0.01760 (18)	
N1	0.50067 (12)	0.82607 (11)	0.33959 (8)	0.0170 (2)	
N2	0.51265 (12)	0.70040 (10)	0.63170 (8)	0.0163 (2)	
C1	0.92887 (18)	1.12337 (16)	0.22198 (11)	0.0293 (3)	
H1A	0.9495	1.1744	0.1652	0.044*	
H1B	0.8424	1.0319	0.1872	0.044*	
H1C	1.0292	1.1051	0.2626	0.044*	
C2	0.87524 (15)	1.21464 (13)	0.30110 (10)	0.0208 (2)	
H2A	0.9631	1.3068	0.3359	0.025*	
H2B	0.7769	1.2364	0.2590	0.025*	
C3	0.83708 (14)	1.14212 (12)	0.38999 (10)	0.0178 (2)	
H3A	0.9302	1.1107	0.4283	0.021*	
H3B	0.8161	1.2092	0.4447	0.021*	
C4	0.63469 (13)	0.93553 (12)	0.39794 (9)	0.0152 (2)	
C5	0.41567 (14)	0.78782 (12)	0.22442 (9)	0.0152 (2)	
C6	0.24671 (14)	0.72731 (12)	0.19297 (10)	0.0176 (2)	
H6	0.1942	0.7184	0.2474	0.021*	
C7	0.15509 (14)	0.68008 (13)	0.08232 (10)	0.0199 (2)	
H7	0.0400	0.6378	0.0612	0.024*	
C8	0.23078 (15)	0.69437 (13)	0.00225 (10)	0.0203 (2)	
H8	0.1678	0.6631	−0.0735	0.024*	
C9	0.39904 (15)	0.75463 (14)	0.03377 (10)	0.0219 (2)	
H9	0.4511	0.7642	−0.0209	0.026*	
C10	0.49257 (14)	0.80118 (13)	0.14456 (10)	0.0197 (2)	
H10	0.6079	0.8417	0.1655	0.024*	
C11	0.15346 (15)	0.40071 (14)	0.77344 (10)	0.0239 (3)	
H11A	0.1401	0.3434	0.8281	0.036*	
H11B	0.0630	0.4418	0.7546	0.036*	
H11C	0.2573	0.4782	0.8040	0.036*	
C12	0.15342 (15)	0.30544 (13)	0.67048 (10)	0.0197 (2)	
H12A	0.2421	0.2610	0.6908	0.024*	
H12B	0.0484	0.2272	0.6404	0.024*	
C13	0.17672 (14)	0.38556 (12)	0.58226 (9)	0.0184 (2)	
H13A	0.1841	0.3213	0.5192	0.022*	
H13B	0.0838	0.4233	0.5555	0.022*	
C14	0.38000 (14)	0.59088 (12)	0.57262 (9)	0.0152 (2)	
C15	0.60105 (13)	0.72986 (12)	0.74636 (9)	0.0151 (2)	
C16	0.66568 (14)	0.87358 (12)	0.80113 (10)	0.0183 (2)	
H16	0.6453	0.9459	0.7630	0.022*	
C17	0.76006 (15)	0.91136 (13)	0.91153 (10)	0.0203 (2)	
H17	0.8049	1.0096	0.9487	0.024*	
C18	0.78895 (14)	0.80571 (13)	0.96760 (10)	0.0204 (2)	
H18	0.8531	0.8313	1.0432	0.025*	
C19	0.72344 (14)	0.66214 (13)	0.91244 (10)	0.0201 (2)	
H19	0.7424	0.5898	0.9509	0.024*	
C20	0.63053 (14)	0.62339 (12)	0.80164 (10)	0.0180 (2)	
H20	0.5877	0.5253	0.7641	0.022*	
H1n	0.452 (2)	0.7739 (17)	0.3777 (13)	0.033 (4)*	
H2n	0.5510 (19)	0.7633 (15)	0.5966 (12)	0.029 (4)*	

### Atomic displacement parameters (Å^2^)

**Table:**

	*U*^11^	*U*^22^	*U*^33^	*U*^12^	*U*^13^	*U*^23^
S1	0.01728 (14)	0.02170 (15)	0.01217 (14)	0.00052 (11)	0.00352 (11)	0.00349 (11)
S2	0.01900 (15)	0.02037 (15)	0.01232 (14)	0.00178 (11)	0.00402 (11)	0.00404 (11)
O1	0.0153 (4)	0.0165 (4)	0.0133 (4)	0.0003 (3)	0.0035 (3)	0.0025 (3)
O2	0.0161 (4)	0.0186 (4)	0.0134 (4)	−0.0014 (3)	0.0034 (3)	0.0037 (3)
N1	0.0150 (4)	0.0206 (5)	0.0132 (5)	0.0010 (4)	0.0050 (4)	0.0042 (4)
N2	0.0173 (5)	0.0166 (5)	0.0138 (5)	0.0023 (4)	0.0046 (4)	0.0051 (4)
C1	0.0328 (7)	0.0362 (7)	0.0263 (7)	0.0113 (6)	0.0175 (6)	0.0128 (6)
C2	0.0217 (6)	0.0192 (5)	0.0188 (6)	0.0016 (4)	0.0056 (5)	0.0065 (5)
C3	0.0173 (5)	0.0159 (5)	0.0151 (5)	−0.0012 (4)	0.0037 (4)	0.0012 (4)
C4	0.0134 (5)	0.0178 (5)	0.0157 (5)	0.0056 (4)	0.0060 (4)	0.0034 (4)
C5	0.0167 (5)	0.0143 (5)	0.0133 (5)	0.0044 (4)	0.0037 (4)	0.0023 (4)
C6	0.0165 (5)	0.0176 (5)	0.0178 (6)	0.0040 (4)	0.0056 (4)	0.0032 (4)
C7	0.0152 (5)	0.0198 (6)	0.0206 (6)	0.0041 (4)	0.0019 (4)	0.0019 (4)
C8	0.0237 (6)	0.0195 (5)	0.0143 (5)	0.0072 (5)	0.0015 (4)	0.0010 (4)
C9	0.0242 (6)	0.0256 (6)	0.0158 (6)	0.0067 (5)	0.0082 (5)	0.0026 (5)
C10	0.0161 (5)	0.0236 (6)	0.0168 (6)	0.0028 (4)	0.0055 (4)	0.0015 (4)
C11	0.0232 (6)	0.0278 (6)	0.0193 (6)	0.0025 (5)	0.0103 (5)	0.0042 (5)
C12	0.0211 (6)	0.0178 (5)	0.0186 (6)	0.0021 (4)	0.0068 (5)	0.0051 (4)
C13	0.0169 (5)	0.0177 (5)	0.0146 (5)	−0.0017 (4)	0.0028 (4)	0.0017 (4)
C14	0.0155 (5)	0.0164 (5)	0.0154 (5)	0.0059 (4)	0.0068 (4)	0.0037 (4)
C15	0.0128 (5)	0.0183 (5)	0.0138 (5)	0.0039 (4)	0.0046 (4)	0.0032 (4)
C16	0.0195 (5)	0.0163 (5)	0.0194 (6)	0.0054 (4)	0.0061 (5)	0.0046 (4)
C17	0.0219 (6)	0.0162 (5)	0.0197 (6)	0.0040 (4)	0.0054 (5)	−0.0003 (4)
C18	0.0193 (6)	0.0243 (6)	0.0152 (5)	0.0057 (5)	0.0035 (4)	0.0024 (5)
C19	0.0201 (5)	0.0207 (6)	0.0188 (6)	0.0071 (4)	0.0036 (5)	0.0063 (5)
C20	0.0179 (5)	0.0152 (5)	0.0188 (6)	0.0046 (4)	0.0037 (4)	0.0025 (4)

### Geometric parameters (Å, º)

**Table:**

S1—C4	1.6745 (12)	C7—H7	0.9500
S2—C14	1.6758 (12)	C8—C9	1.3869 (17)
O1—C4	1.3298 (14)	C8—H8	0.9500
O1—C3	1.4596 (13)	C9—C10	1.3920 (16)
O2—C14	1.3280 (14)	C9—H9	0.9500
O2—C13	1.4543 (13)	C10—H10	0.9500
N1—C4	1.3412 (15)	C11—C12	1.5213 (17)
N1—C5	1.4233 (14)	C11—H11A	0.9800
N1—H1n	0.881 (9)	C11—H11B	0.9800
N2—C14	1.3370 (15)	C11—H11C	0.9800
N2—C15	1.4277 (15)	C12—C13	1.5090 (16)
N2—H2n	0.876 (9)	C12—H12A	0.9900
C1—C2	1.5230 (18)	C12—H12B	0.9900
C1—H1A	0.9800	C13—H13A	0.9900
C1—H1B	0.9800	C13—H13B	0.9900
C1—H1C	0.9800	C15—C20	1.3908 (16)
C2—C3	1.5096 (16)	C15—C16	1.3909 (16)
C2—H2A	0.9900	C16—C17	1.3893 (17)
C2—H2B	0.9900	C16—H16	0.9500
C3—H3A	0.9900	C17—C18	1.3884 (17)
C3—H3B	0.9900	C17—H17	0.9500
C5—C10	1.3931 (16)	C18—C19	1.3910 (17)
C5—C6	1.3928 (16)	C18—H18	0.9500
C6—C7	1.3868 (16)	C19—C20	1.3901 (16)
C6—H6	0.9500	C19—H19	0.9500
C7—C8	1.3891 (18)	C20—H20	0.9500
			
C4—O1—C3	118.40 (9)	C10—C9—H9	119.6
C14—O2—C13	119.24 (9)	C9—C10—C5	119.44 (11)
C4—N1—C5	129.52 (10)	C9—C10—H10	120.3
C4—N1—H1n	116.3 (11)	C5—C10—H10	120.3
C5—N1—H1n	113.9 (12)	C12—C11—H11A	109.5
C14—N2—C15	128.33 (10)	C12—C11—H11B	109.5
C14—N2—H2n	116.9 (11)	H11A—C11—H11B	109.5
C15—N2—H2n	114.8 (11)	C12—C11—H11C	109.5
C2—C1—H1A	109.5	H11A—C11—H11C	109.5
C2—C1—H1B	109.5	H11B—C11—H11C	109.5
H1A—C1—H1B	109.5	C13—C12—C11	113.18 (10)
C2—C1—H1C	109.5	C13—C12—H12A	108.9
H1A—C1—H1C	109.5	C11—C12—H12A	108.9
H1B—C1—H1C	109.5	C13—C12—H12B	108.9
C3—C2—C1	112.87 (11)	C11—C12—H12B	108.9
C3—C2—H2A	109.0	H12A—C12—H12B	107.8
C1—C2—H2A	109.0	O2—C13—C12	106.33 (9)
C3—C2—H2B	109.0	O2—C13—H13A	110.5
C1—C2—H2B	109.0	C12—C13—H13A	110.5
H2A—C2—H2B	107.8	O2—C13—H13B	110.5
O1—C3—C2	107.03 (9)	C12—C13—H13B	110.5
O1—C3—H3A	110.3	H13A—C13—H13B	108.7
C2—C3—H3A	110.3	O2—C14—N2	112.77 (10)
O1—C3—H3B	110.3	O2—C14—S2	124.50 (9)
C2—C3—H3B	110.3	N2—C14—S2	122.71 (9)
H3A—C3—H3B	108.6	C20—C15—C16	120.28 (11)
O1—C4—N1	112.98 (10)	C20—C15—N2	123.06 (10)
O1—C4—S1	124.75 (8)	C16—C15—N2	116.58 (10)
N1—C4—S1	122.27 (9)	C17—C16—C15	120.03 (11)
C10—C5—C6	119.92 (11)	C17—C16—H16	120.0
C10—C5—N1	123.81 (10)	C15—C16—H16	120.0
C6—C5—N1	116.20 (10)	C16—C17—C18	120.08 (11)
C7—C6—C5	120.09 (11)	C16—C17—H17	120.0
C7—C6—H6	120.0	C18—C17—H17	120.0
C5—C6—H6	120.0	C17—C18—C19	119.60 (11)
C6—C7—C8	120.30 (11)	C17—C18—H18	120.2
C6—C7—H7	119.9	C19—C18—H18	120.2
C8—C7—H7	119.9	C20—C19—C18	120.74 (11)
C9—C8—C7	119.50 (11)	C20—C19—H19	119.6
C9—C8—H8	120.2	C18—C19—H19	119.6
C7—C8—H8	120.2	C19—C20—C15	119.27 (11)
C8—C9—C10	120.75 (11)	C19—C20—H20	120.4
C8—C9—H9	119.6	C15—C20—H20	120.4
			
C4—O1—C3—C2	179.25 (10)	C14—O2—C13—C12	179.29 (10)
C1—C2—C3—O1	−66.95 (13)	C11—C12—C13—O2	55.92 (13)
C3—O1—C4—N1	179.65 (9)	C13—O2—C14—N2	174.58 (9)
C3—O1—C4—S1	−0.49 (14)	C13—O2—C14—S2	−4.13 (15)
C5—N1—C4—O1	0.59 (17)	C15—N2—C14—O2	0.88 (17)
C5—N1—C4—S1	−179.27 (9)	C15—N2—C14—S2	179.63 (9)
C4—N1—C5—C10	37.09 (18)	C14—N2—C15—C20	39.15 (18)
C4—N1—C5—C6	−146.20 (12)	C14—N2—C15—C16	−144.15 (12)
C10—C5—C6—C7	0.15 (17)	C20—C15—C16—C17	−0.04 (17)
N1—C5—C6—C7	−176.70 (10)	N2—C15—C16—C17	−176.84 (10)
C5—C6—C7—C8	−0.78 (18)	C15—C16—C17—C18	−0.51 (18)
C6—C7—C8—C9	0.79 (18)	C16—C17—C18—C19	0.26 (18)
C7—C8—C9—C10	−0.17 (19)	C17—C18—C19—C20	0.53 (18)
C8—C9—C10—C5	−0.46 (19)	C18—C19—C20—C15	−1.07 (18)
C6—C5—C10—C9	0.47 (18)	C16—C15—C20—C19	0.82 (17)
N1—C5—C10—C9	177.06 (11)	N2—C15—C20—C19	177.41 (10)

### Hydrogen-bond geometry (Å, º)

**Table:**

*D*—H···*A*	*D*—H	H···*A*	*D*···*A*	*D*—H···*A*
N1—H1*n*···S2	0.88 (1)	2.51 (1)	3.3667 (10)	164 (2)
N2—H2*n*···S1	0.88 (1)	2.52 (1)	3.3765 (10)	167 (2)
